# Endocytosis Deficient Murine Xcl1-Fusion Vaccine Enhances Protective Antibody Responses in Mice

**DOI:** 10.3389/fimmu.2019.01086

**Published:** 2019-05-17

**Authors:** Arnar Gudjonsson, Tor Kristian Andersen, Vibeke Sundvold-Gjerstad, Bjarne Bogen, Even Fossum

**Affiliations:** ^1^K.G. Jebsen Centre for Influenza Vaccine Research, Institute of Immunology, Oslo University Hospital, University of Oslo, Oslo, Norway; ^2^Department of Molecular Medicine, Institute of Basic Medical Sciences, University of Oslo, Oslo, Norway; ^3^Centre for Immune Regulation, Institute of Immunology, Oslo University Hospital, University of Oslo, Oslo, Norway

**Keywords:** targeting, Xcl1, cDC1 dendritic cells, vaccine, DNA vaccine, antibody response

## Abstract

Targeting antigen to surface receptors on dendritic cells (DCs) can improve antibody response against subunit vaccines. We have previously observed that human XCL1-fusion vaccines target murine Xcr1^+^ DCs without actively inducing endocytosis of the antigen, resulting in enhanced antibody responses in mice. However, the use of foreign chemokines for targeting is undesirable when translating this observation to human or veterinary medicine due to potential cross-reactive responses against the endogenous chemokine. Here we have identified a mutant version of murine Xcl1, labeled Xcl1(Δ1) owing to removal of a conserved valine in position 1 of the mature chemokine, that retains specific binding to Xcr1^+^ DCs without inducing endocytosis of the receptor. DNA immunization with Xcl1(Δ1) conjugated to influenza hemagglutinin (HA) induced improved antibody responses, with higher end point titers of IgG compared to WT Xcl1-HA. The Xcl1(Δ1) fusion vaccine also resulted in an increased number of HA reactive germinal center B cells with higher avidity toward the antigen, and serum transfer experiments show that Xcl1(Δ1)-HA induced antibody responses provided better protection against influenza infection as compared to WT Xcl1-HA. In summary, our observations indicate that targeting antigen to Xcr1^+^ DCs in an endocytosis deficient manner enhances antibody responses. This effect was obtained by introducing a single mutation to Xcl1, suggesting our strategy may easily be translated to human or veterinary vaccine settings.

## Introduction

Targeting antigen to antigen-presenting cells such as DCs, with the intention of improving efficacy of subunit vaccines has shown great promise in pre-clinical studies (1, 2). Although the focus of this strategy has mainly been to improve T cell responses, it has also been shown to efficiently enhance antibody responses by others (3–6) and by us (7–9). However, the mechanisms that lead to enhanced antibody responses when targeting DCs are still unclear, and may even differ depending on the surface receptor being targeted (10–15).

The chemokine receptor Xcr1 is selectively expressed on type 1 conventional dendritic cells (cDC1s) in mice (16, 17), and this selective expression appears to be conserved in man (18–20) as well as in other mammals such as sheep (18), pigs (21), and macaques (22). To selectively deliver antigen to the Xcr1 receptor we have previously used the chemokine Xcl1 as a targeting unit (12, 23). In a recent study, we observed that targeting the Xcr1^+^ cDC1 population without actively inducing endocytosis of the antigen resulted in improved antibody responses, both with regards to endpoint titers and protective ability during infection (9). This study was, however, performed using human XCL1 and XCL2 chemokines as targeting units in mice, which raises issues. First, it would be problematic to use foreign chemokines in human or veterinary medicine, as these could result in breaking of tolerance toward the endogenous chemokine. Second, it is possible that the human XCL1 and XCL2 contain helper epitopes that increase the immunogenicity of the fusion vaccines which would be absent when using endogenous chemokines. To resolve these issues, we set out to identify a fully murine Xcl1 mutant that could be used to target Xcr1^+^ DCs without inducing receptor-mediated endocytosis and determine whether it too would improve antibody responses when fused to antigen. While it is not known how the Xcl1 chemokine interacts with the Xcr1 receptor, the N-terminal part of Xcl1 has been reported to be important for receptor activation (24). This is in line with published models for chemokine/receptor interaction, where the chemokine binds and activates the receptor through a two-step mechanism involving the N-terminus of the chemokine (25–27). With this in mind, we focused on the N-terminal part of Xcl1 when introducing mutations, before evaluating them for binding and endocytosis. While N-terminal substitutions failed to generate mutants with the desirable behavior, removing the first N-terminal amino acid from the mature chemokine resulted in a mutant [labeled Xcl1(Δ1)] that retained binding to Xcr1^+^ DCs but did not induce endocytosis. Here, we have used a murine influenza model to show that immunization with a fusion vaccine containing Xcl1(Δ1) resulted in improved antibody responses that provided better protection against infection.

## Materials and Methods

### Mice

Female BALB/cAnNRj mice were obtained from Janvier Labs, France. Twenty percent weight loss after viral challenge was set as a humane endpoint, in accordance with the guidelines of the Norwegian Animal Research Authority. Xcr1 knockout mice, B6.129P2-Xcr1^tm1Dgen^, were obtained from the Jackson Laboratory, USA.

### Generation of Mutant Vaccibodies

Cloning of vaccibodies was done as previously reported (9). In short, the vector pLNOH2, derived from pcDNA3 (Invitrogen) (28), was used. Sequences encoding the mutant versions of murine Xcl1 were obtained from GenScript with added 5′ BsmI and 3′ BsiWI sites and cloned into vectors expressing HA or mCherry.

### Expression and Purification of Vaccibody Proteins

For purification of mCherry-vaccibody proteins, 80% confluent HEK293E cells in five-layer BD Falcon Multi-flasks (Corning; Life Sciences, Durham, CA) were transfected using Polyethylenimine (PEI) complexed DNA. Supernatant from transfected cells was harvested after 4–5 days and run through a CaptureSelect^TM^ FcXL affinity column (Thermo Fisher) specific for the vaccibody dimerization domain, with an ÄKTAprime plus chromatography system (GE Healthcare, Wauwatosa, WI). For *in vitro* expression of HA-vaccibodies, 80% confluent HEK293E cells in 6-well plates were transfected using PEI.

### ELISA

For all ELISAs, High-binding 96-well plates (Costar, Corning, NY) were used. All ELISAs were developed with phosphatase substrate (Sigma) and OD was measured at 405 nm with a TECAN microplate reader or a AlphaScreen.

#### ELISA for Detecting Anti-HA IgG Responses in Serum

Plates were coated with inactivated PR8 virus (2 μg/ml) in PBS (0.02% w/v NaAzide) over night (ON) at 4°C. Plates were incubated for 1.5 h at RT with either ALP-conjugated anti-mouse IgG (Fc-specific, 1:5,000), or biotinylated anti-mouse IgG1(a), IgG2a(a), or IgG2b and subsequently streptavidin-alkaline phosphatase (1:3,000, Sigma), and developed with phosphatase substrate for 30 min. Ab endpoint titer was defined as the last dilution of a sample with an OD value higher than mean + 3 × SD of the same dilution from serum from NaCl vaccinated mice. If OD did not develop above that of NaCl mice (mean + 3 × SD), the sample was given an arbitrary value of 10 in endpoint titer plots, representing a non-detectable titer.

#### ELISA for Detecting Secretion of Vaccine Protein

Plates were coated with mouse anti-human CH3 domain (MCA878G, Sigma) in PBS (0.02% w/v NaAzide) ON at 4°C. Supernatants from transfected cells were incubated ON at 4°C or for a minimum of 1 h at RT, plates were then washed and vaccibody proteins were detected using anti-HA mAb (clone H36-4-52).

### DNA Immunization and Electroporation

The mice were anesthetized with 150 μl (no more than 0.01 mg/g bodyweight) of ZRF mixture consisting of 250 mg/ml Zoletil Forte (Virbac), 20 mg/ml Rompun (Bayer Animal Health), and 50 μg/ml Fentanyl (Actavis). The lower dorsal region was shaved and injected intradermally (i.d.) with 25 μl of saline solution containing the DNA vaccine (0.5 mg/ml DNA in NaCl) on one side of the lower flank, the injection site was immediately electroporated (Two pulses of 450 V/cm x 2.5 μs and eight pulses of 110 V/cm × 8.1 ms) using a needle array electrode and a DermaVax (BTX Harvard Apparatus, Holliston, MA). The procedure was repeated for the other flank, and each mouse received a total of 25 μg DNA.

### IFN-γ ELISPOT

Splenocytes were prepared using the GentleMACS dissociator according to the manufacturer's enzyme-free protocol, and ELISpotPLUS for Mouse IFN-γ kit with precoated anti-IFN-γ plates were used in accordance to the manufacturer's protocol (Mabtech AB, Nack Straand, Sweden). In short, spleens were crushed and treated with tris-buffered ammonium chloride (ACT) erythrocyte lysis buffer and filtered through a 75 μm Nylon strainer. The single cell suspension was then seeded in the plates at a concentration of 0.5 × 10^6^ per well and re-stimulated with 2 μg/ml of HA-derived peptides IYSTVASSL (MHCI), HNTNGVTAACSHEG (MHCII) for 18 h at 37°C 5% CO_2_. The plates were then automatically counted and analyzed with a CTL ELISPOT reader (CTL Europe, Bonn, Germany).

### Influenza Virus Challenge

Prior to infection, the virus dose that is lethal to 50% of naïve mice, the LD50 dose, was determined. Actively and passively immunized mice were anesthetized with ZRF mixture before receiving 10 μl of virus in PBS in each nostril, and observed until they had inhaled the liquid. Mice were weighed daily as a measure of disease progression and were euthanized if they lost more than 20% of original weight. In weight curves, euthanized mice are plotted as having a value of 80% throughout the rest of the experiment.

### Microneutralization Assay

Costar 96 well-cell culture plates were used. Sera from mice were diluted 1:3 in receptor destroying enzyme (RDE) solution (Denka Seiken co, Tokyo, Japan) and incubated ON at 37°C before they were heat-inactivated at 56°C for 30 min. Forty microliter of virus diluent [40 ml Gibco DMEM, 0.48 ml M.T, 1 ml 1 M HEPES, 4.64 ml Fraction V (10%)] was added per well in the top row, and 50 μl in the rest. Sixty microliter of heat-inactivated sera were added per well in the top row, and diluted 2-fold for each row. Fifty microliter of diluent containing virus [100 × tissue culture infection dose (TCID50 previously determined)] was added per well. This mixture was incubated for 2 h at 37°C. Madin Darby canine kidney (MDCK) epithelial cells were plated at a concentration of 20.000 cells in 100 μl virus diluents per well-before the plates were incubated ON at 37°C and 5% CO_2_. The plates were then fixed by removing medium, washing with PBS and incubation with cold fixative (80% acetone) for 10 min before they were air dried. ELISA was performed after the plates were washed 3 times with wash buffer (0.3% TWEEN 20 in PBS). Plates were incubated for 1 h at RT with anti-nucleo protein (NP) H16-L10-4R5 biotin antibody (1:1,000, 0.7 μg/ml, in ELISA buffer). Next, plates were washed and incubated for 1 h at RT with streptavidin ALP (Sigma), washed, and developed with 100 μl of ALP substrate. After ~20 min, OD405 was measured on a TEACAN microplate reader. Negative values were given a value of 0.

### Serum Transfer

Immunized mice were anesthetized with ZRF mixture and drained of blood by heart puncture 6 weeks after immunization or 3 weeks after boost. Sera from each group were pooled, and 300 μl serum was injected intraperitoneally (i.p.) to naïve mice 1 day before being challenged as described above.

### T Cell Depletion

Mice were injected i.p. with a mix of 100 μg anti-CD8 [TIB105(53.6.72)] and 100 μg anti-CD4 (GK1.5), or isotype control on day −3, 0, and 3 relative to viral challenge. Spleens were harvested from mice receiving the same injections 3 days after injection and evaluated by flow cytometry for depletion efficacy using anti-CD3e (145-2C11; Tonbo Biosciences), anti-CD45R (RA3-6B2; Tonbo Biosciences), anti-CD4 (1540-11; Southern Biotech) and anti-CD8 (553033; BD Biosciences) and isotype controls.

### DC Isolation From Spleen

DCs from spleens of BALB/c mice were prepared using the GentleMACS dissociator (Miltenyi Biotech) according to the manufacturer's protocol. Briefly, spleens were dissociated in GentleMACS C tubes in medium containing collagenase and DNase, incubated for 15 min at 37 C before adding EDTA at a final concentration of 10 mM. Erythrocytes were lysed by incubation with ACT buffer for 5 min on ice. Finally, cells were filtered through a 75 mm Nylon cell strainer. The following Abs were used for subsequent flow cytometry analysis: anti-CD3e (145-2C11; Tonbo Bio- sciences), anti-CD19 (1D3; Tonbo Biosciences), anti-CD49b (DX5; eBioscience), anti-Ly6G (1A8), anti-CD45R (RA3-6B2; Tonbo Biosciences), anti-MHC-II (M5/114.15.2; BioLegend), anti-CD11c (N418; Tonbo Bio- sciences), anti-CD11b (M1/70; Tonbo Biosciences), and anti-CD24 (M1/69; BioLegend).

### *In vitro* Generation of Flt3L DCs

Bone marrow cells were harvested by flushing tibia and femur with RPMI medium with 10% FCS. The cell suspension was filtered through a 75 μm Nylon cell strainer, seeded at a concentration of 2 × 10^6^ cells/ml, 5 ml/well in a 6-well plate. Then, 0.1 μg/ml of Flt3L (Peprotech, NJ) was added and the cells were incubated for 9 days at 37°C 5% CO_2_ (29). Semi-adherent cells were subsequently harvested and analyzed by flow cytometry after staining with anti-CD45R/B220 (RA3-6B2, Tonbo Biosciences), anti-CD11c (N418, Tonbo Biosciences), anti-CD11b (M1/70, Tonbo Biosciences), and anti-CD24 (M1/69, BioLegend) for 20 min on ice. For staining with mCherry vaccibodies, the cells were subsequently incubated with purified vaccibody proteins at a concentration of 20 μg/ml for 25 min on ice.

### Chemotaxis on Flt3L DCs

Flt3L DCs were added to the upper wells of a 24 well-Transwell plate (Costar) at a concentration of 1 × 10^6^ per well. In the lower wells, purified mCherry vaccibody proteins were used at a concentration of 1,500 ng/ml. Cells were incubated for 4 h at 37°C 5% CO_2_ before cells in the lower chamber were harvested, stained for 20 min with anti-CD45R/B220, anti-CD11c, anti-CD11b, anti-CD24, and analyzed by flow cytometry.

### Endocytosis Assay

Flt3L DCs were incubated with supernatant from transfected cells or purified protein on ice for 30 min, washed, and incubated at 37°C 5% CO2 for 0, 15, or 30 min. Next, the cells were stained with anti-CD45R/B220, anti-CD11c, anti-CD11b, anti-CD24, and biotinylated anti-mCherry for 20 min, washed, and incubated with streptavidin- allophycocyanin-Cy7 conjugate (405208; BioLegend) for 15 min. The cells were subsequently analyzed by flow cytometry. Internalization was defined as the MFI signal ratio of APC-Cy7 to mCherry relative to *t* = 0 min. The ratio at *t* = X is divided by the ratio at *t* = 0 giving the values plotted. For *t* = 0 the value is 1.

### Flow Cytometry

All flow cytometry experiments except GC B cell experiments were performed on a LSRFortessa flow cytometer (BD biosciences) and analyzed using FlowJo 10.0.8 software. Compensations were performed using eComp beads (eBiosciences). Forward light scatter A vs. forward light scatter H and side scatter A vs. side scatter H were used for doublet exclusion in all assays.

### ImageStream Method

Data of 5 × 10^4^ cells per sample were acquired on a 12-channel ISX Imaging Flow Cytometer with 403 objective (Amnis). Single stained controls were collected with bright-field illumination off, and with all necessary excitation lasers switched on. A compensation matrix was created using single stained raw image files and the IDEAS compensation wizard. The matrix was used to compensate the raw sample files to correct for spectral overlap. The data were analyzed using IDEAS 6.1 software (Amnis). Single cells were identified by a bright field area vs. bright field aspect ratio plot. Cells in focus were identified using the gradient root mean square feature of the bright field image. Viable cells were identified using bright field contrast to threshold of the nucleus area plot. The intensity feature was used to identify the Xcr1^+^ cells (Channel 11, anti-Xcr1 APC) and mCherry^+^ cells (Channel 3). Intracellular localization of Xcr1 was measured with the internalization feature, which is the ratio of the intensity inside the cell to the intensity of the entire cell.

### Detection of HA Reactive GC B Cells

Mice were DNA vaccinated as described previously and draining LNs (inguinal) were harvested 3, or 5 weeks later. Single cell suspensions were prepared by GentleMACS dissociator. Recombinant HA (PR8) with tyrosine substituted with phenylalanine at position 98 (Y98F) (30) and a carboxy terminal 6xhistidine tag was affinity purified in the laboratory. GC B cells were defined as CD3^−^B220^+^CD38^lo^GL7^+^ stained with anti-CD3 (75-0032), anti-GL7 (144603), anti-CD38 (102718) from Tonbo biosciences, San Diego, CA, USA, anti-B220 (552771) from BD Biosciences, Franklin Lakes, NJ, USA, and anti-6x His tag (ab133714) from Abcam, Cambridge, England. All setups included appropriate fluorescence minus one with fluorochrome matched isotype control. All samples were analyzed using an Attune NxT flow cytometer (Thermo Fisher Scientific, Waltham, MA, USA) and FlowJo software.

### Curve Fitting and Statistical Analysis

Antigen binding dilution curves obtained in flow cytometry was fitted with a one-site total binding least squares fit. Background was constrained to the average value detected against the irrelevant antigen mCherry. Statistical significance of fitted values was calculated by extra sum of squares test. For binding, endocytosis, ELISPOT and quantifying of GC B cells, unpaired *t*-test (two-tailed) were used. For serum endpoint titers, Mann-Whitney (two-tailed) was performed. For analysis of weight curves and neutralizing titer curves, two-way ANOVAs witch Tukey's multiple comparisons test were done. Mantel-Cox was performed for the survival curve. All analysis was performed using GraphPad Prism 6 software.

### Study Approval

All *in vivo* studies were pre-approved by the Norwegian Animal Research Authority, and performed in compliance with their guidelines.

## Results

### Murine Xcl1(Δ1) Binds Xcr1^+^ cDC1s, but Is Not Actively Endocytosed

**Figure 1:**
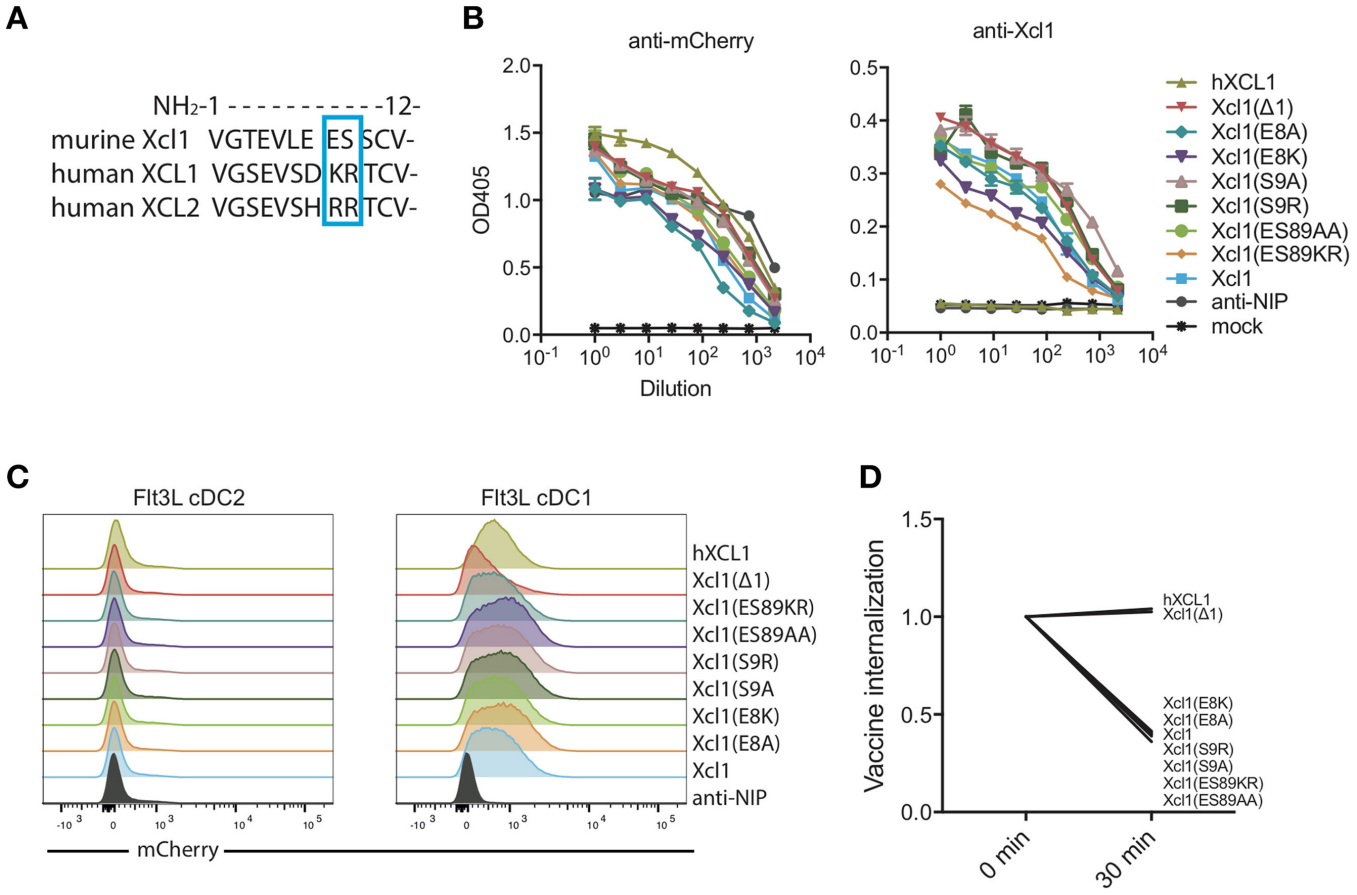
Screening for a murine Xcl1 mutant that binds cDC1s but is not endocytosed. **(A)** Sequence alignment for the first 12 N-terminal amino acids of murine Xcl1 and human XCL1 and XCL2. The blue square indicates the positions at which the mutations were done, positions 8 and 9. **(B)** ELISA of supernatants from transfected HEK293E cells with the different vaccibody constructs. Coating antibody: anti-Human CH3 domain (dimerization unit). Detection antibody: anti-mCherry (left) or murine anti-Xcl1 (right). **(C)** BM-derived Flt3L-induced cDC1s (right) and cDC2s (left) were gated as presented in Supplementary Figure 1B, and evaluated for binding of the different vaccibodies by incubation with supernatant from transfected cells. **(D)** Internalization of vaccibodies by Flt3L cDC1s was determined by flow cytometry. After staining with supernatants containing mCherry vaccibodies, cells were incubated on ice or at 37°C for 30 min before they were stained with biotinlyated anti-mCherry and streptavidin-APC-Cy7 to stain surface bound vaccibodies. The change in the MFI signal ratio of the surface bound vaccibody signal (APC-Cy7) to total vaccibody signal (mCherry) is plotted. On the x-axis, time points indicate incubation time at 37°C. **(B)** Data shown are mean ± SEM. (B) *n* = 3 **(C)** data shown are from one representative experiment of at least two experiments. **(D)** Data are pooled from three different experiments, values shown are mean, *n* = 2–3.

We aimed to identify a mutated murine Xcl1 that retained its specificity toward Xcr1^+^ DCs but lost the ability to activate the receptor and be actively endocytosed. Based on our previous observation that human XCL1 and XCL2 bound murine Xcr1^+^ DCs without inducing endocytosis (9), we used sequence differences between the human and murine chemokines as a starting point for introducing mutations. We performed alanine substitutions in the N-terminal region of the protein at positions 8 and 9, which are occupied by the basic amino acids lysine (K) and arginine (R) in XCL1 and two arginines in XCL2. In contrast, the murine Xcl1 has an acidic glutamic acid (E) and hydroxylic serine (S) in position 8 and 9, respectively (Figure 1A). We also replaced the WT murine amino acids E8 and S9 with the human XCL1 K8 and R9. Lastly, we made a mutant that lacked the first N-terminal amino acid, Xcl1(Δ1), as this mutation has previously been reported to result in loss of receptor activation for human XCL1 (24).

To screen for binding and endocytosis, we generated bivalent fusion vaccine molecules (vaccibodies) containing each of these mutants as targeting units, and the fluorescent protein mCherry in the antigenic unit (Supplementary Figure 1A). All constructs were found to be expressed at comparable levels when transfected into HEK293E cells, both when detected with anti-mCherry and anti-Xcl1 in ELISA (Figure 1B). To evaluate binding and endocytosis, the mutant Xcl1-mCherry supernatants were incubated with bone marrow derived Flt3L DCs for 30 min on ice, washed and subsequently incubated either on ice or at 37°C for 30 min to allow for receptor mediated endocytosis. Flt3L derived cDC1s were defined as CD45R^−^CD11c^+^CD24^+^ cells (Supplementary Figure 1B). Surface-bound mCherry vaccibodies were identified with biotinylated anti-mCherry and streptavidin APC-Cy7. The APC-Cy7 to mCherry MFI signal ratio was around 2 for all constructs at *t* = 0 (data not shown), but after endocytosis of the constructs the APC-Cy7 signal drops resulting in a drop in the ratio. As a non-targeted control, a fluorescent mCherry vaccibody construct containing a single chain variable fragment specific for the hapten NIP was included (referred to as anti-NIP-mCherry) (7), and the previously published human XCL1 (hXCL1) mCherry vaccibody construct was used as a positive control (9). Somewhat surprisingly, all our substitution mutants bound and were actively internalized to the same degree as WT Xcl1. In contrast, the Δ1 mutant specifically bound the cDC1 population but failed to be internalized, behaving like the human hXCL1 (Figures 1C,D).

**Figure 2:**
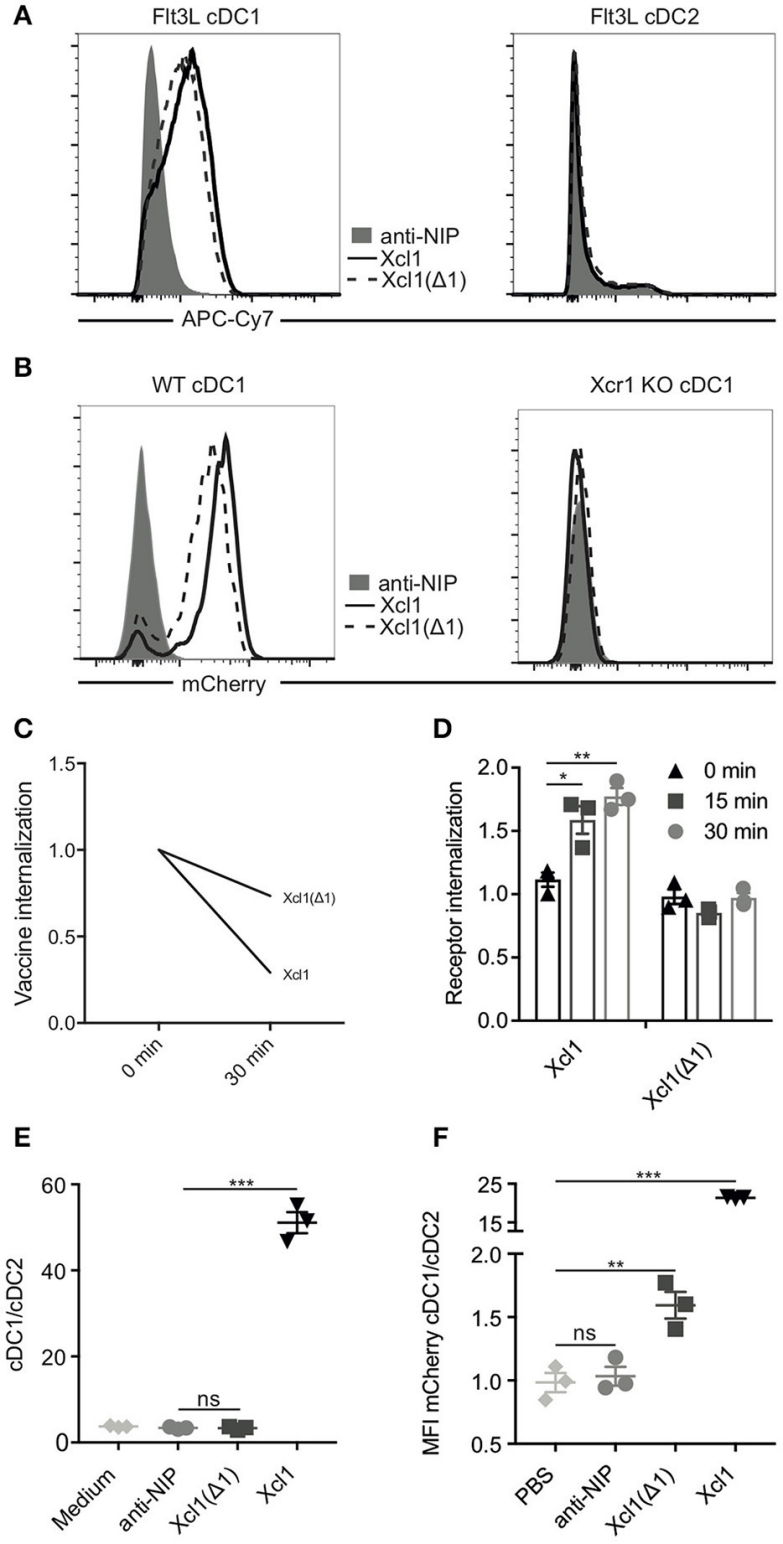
Xcl1(Δ1) specifically binds cDC1s, but is not actively internalized. **(A)** BM-derived Flt3L-induced cDC1s (left) and cDC2s (right) from BALB/c mice were analyzed by flow cytometry for binding to Xcl1-mCherry and Xcl1(Δ1)-mCherry vaccibodies with anti-NIP-mCherry as a negative control. mCherry vaccibodies were detected with biotinlyated anti-mCherry mAb and streptavidin-APC-Cy7 to boost the signal. **(B)** cDC1s from WT or Xcr1 KO mouse splenocytes were gated as Lin^−^MHCII^+^CD11c^+^ and CD24^+^ (cDC1s) or CD11b^+^ (cDC2s) as shown in Supplementary Figure 1C, and evaluated for binding to mCherry vaccibodies. **(C)** Internalization of purified mCherry vaccibodies by Flt3L DCs was determined as described in Figure 1D. **(D)** Internalization of the Xcr1 receptor on Flt3L DCs after incubation with Xcl1- or Xcl1(Δ1)-mCherry vaccibodies for 0, 15, or 30 min at 37°C as determined by ImageStream. The plotted values are relative to background internalization after incubation with anti-NIP-mCherry vaccibodies. **(E)** Migration of Flt3L DCs in transwell plates after incubation with mCherry vaccibody proteins. Ratio of cDC1 and cDC2 was determined by flow cytometry. **(F)** *In vivo* binding of mCherry vaccibody proteins as determined by flow cytometry. Spleens were harvested 1 h after i.v. injection of 25 μg of protein, and splenic DCs were defined as in **(B)**. **(C)** Data are pooled from two separate experiments, *n* = 4, values shown are mean. **(D–F)** Data shown are mean ± SEM. **(D)** Data are pooled from three separate experiments, *n* = 3. **(E,F)** *n* = 3. **(D–F)** Unpaired *t-*test (two-tailed), *p < 0.05, **p < 0.01, ***p < 0.001.

Based on these results we produced and purified Xcl1(Δ1)-mCherry vaccibody protein. This protein was confirmed to be specific for Flt3L cDC1s by flow cytometry (Figure 2A; Supplementary Figure 1B). To enhance the signal, biotinylated anti-mCherry was used in combination with streptavidin APC-Cy7. Binding was lost on cDC1 (defined as Lin^−^MHC-II^+^CD11c^+^CD24^+^) obtained from Xcr1 knockout mice, confirming specificity for the Xcr1 receptor (Figure 2B; Supplementary Figure 1C). As expected, the purified Xcl1(Δ1)-mCherry protein also endocytosed to a lesser extent than WT Xcl1-mCherry (Figure 2C). To ensure that the Xcr1 receptor itself indeed remained on the surface after incubation with Xcl1(Δ1)-mCherry, Flt3L DCs were stained with an anti-Xcr1 ab as well as with WT and mutant Xcl1-mCherry proteins, washed and subsequently incubated on ice or 37°C for 15 or 30 min followed by analysis for endocytosis by Imagestream. As previously shown, incubation with WT Xcl1 resulted in internalization of the Xcr1 receptor (Figure 2D) (9). Incubation with Xcl1(Δ1)-mCherry, however, did not induce internalization of the receptor. These results suggest that that the Xcl1(Δ1) mutant lacks the ability to activate the Xcr1 receptor. To further test this hypothesis, we performed a chemotaxis assay where Flt3L DCs were added to the upper compartment of a transwell plate, while anti-NIP-, WT-, or Xcl1(Δ1)-mCherry proteins were added to the bottom compartment. The cDC1/cDC2 ratio of the migrated cells was determined by flow cytometry and, as expected, only incubation with WT Xcl1-mCherry resulted in specific migration of cDC1s (Figure 2E). To ensure that the Xcl1(Δ1) mutant is able to target cDC1s *in vivo*, 25 μg of protein was injected i.v. and spleens were harvested after 1 h. Both WT and the mutant specifically bound to the cDC1 population, as analyzed by flow cytometry. In accordance with our *in vitro* endocytosis results, the increased MFI seen with the WT compared to the mutant Xcl1-mCherry could reflect accumulation of protein due to active endocytosis *in vivo* (Figure 2F). Taken together, these data show that the Xcl1(Δ1) mutant specifically binds Xcr1 on cDC1s, but unlike the WT, is not endocytosed by activating the receptor.

### Xcl1(Δ1) Targeting Enhances Antibody Responses After i.d. DNA Immunization

**Figure 3:**
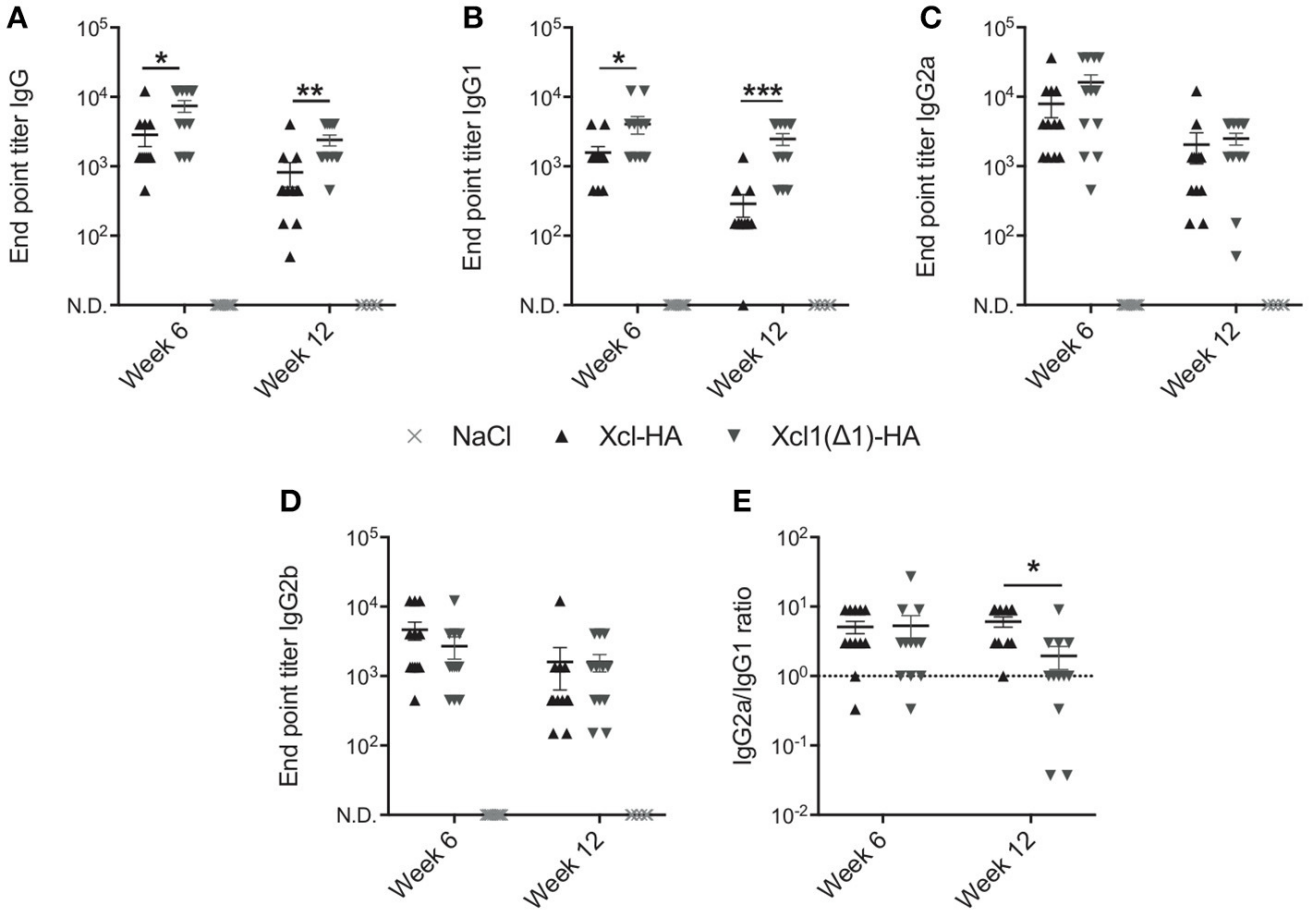
Xcl1(Δ1)-targeting augments antibody responses. Six to eight week old BALB/c mice were vaccinated once with intradermal injection of DNA plasmids encoding Xcl1- or Xcl1(Δ1)-HA vaccibodies, or saline solution, followed by electroporation of the injection site. Serum samples were harvested after 6 and 12 weeks and evaluated for endpoint titers of HA-specific **(A)** Total IgG, **(B)** IgG1, **(C)** IgG2a, and **(D)** IgG2b. **(E)** IgG2a/IgG1 ratio. **(A–E)** Data shown are mean ± SEM. For week 6, data are pooled from two separate experiments, *n* = 12 for vaccine groups, for NaCl *n* = 6. For week 12, *n* = 12 for vaccine groups, for NaCl *n* = 6. **(A–E)** Mann-Whitney (two-tailed), *p < 0.05, **p < 0.01, ***p < 0.001.

The above results encouraged us to compare WT Xcl1 to Xcl1(Δ1) in the context of cDC1 targeting *in vivo* to see if the latter would improve antibody responses as we hypothesize. We have previously demonstrated the benefits of targeting cDC1s using Xcl1 vaccibodies compared to non-targeted controls, and therefore focus on comparing WT Xcl1 and Xcl1(Δ1) in the present study (9, 12). BALB/c mice were vaccinated once intradermally with 25 μg DNA plasmids encoding vaccibodies with Xcl1(Δ1) or WT Xcl1 as targeting units and hemagglutinin (HA) from influenza A/PR/8/34 (PR8) as antigen. After intradermal delivery of DNA, the injection site was electroporated (EP) to facilitate uptake of the plasmids and enhance protein expression (31). Negative control mice received saline solution followed by electroporation. Serum samples were harvested after 6 and 12 weeks and HA specific total IgG was determined by ELISA. In accordance with our hypothesis, Xcl1(Δ1) induced significantly higher titers of HA specific IgG after both 6 and 12 weeks compared to Xcl1-HA (Figure 3A). Furthermore, Xcl1(Δ1)-HA induced significantly higher titers of IgG1 compared to Xcl1-HA at both time points (Figure 3B). No significant differences were observed for IgG2a or IgG2b, although there was a slight tendency for higher IgG2a and lower IgG2b titers with Xcl1(Δ1)-HA at week 6 (Figures 3C,D). Interestingly, we only observed a difference in the IgG2a/IgG1 ratio between Xcl1-HA and Xcl1(Δ1)-HA at week 12 after immunization (Figure 3E). Thus, the lack of receptor activation and internalization with Xcl1(Δ1) results in a shift in the antibody polarization toward IgG1 with time, compared to targeting with the WT chemokine (Figure 3E).

### Xcl1(Δ1)-HA Immunization Results in Reduced CD8^+^ T-Cell Responses

**Figure 4:**
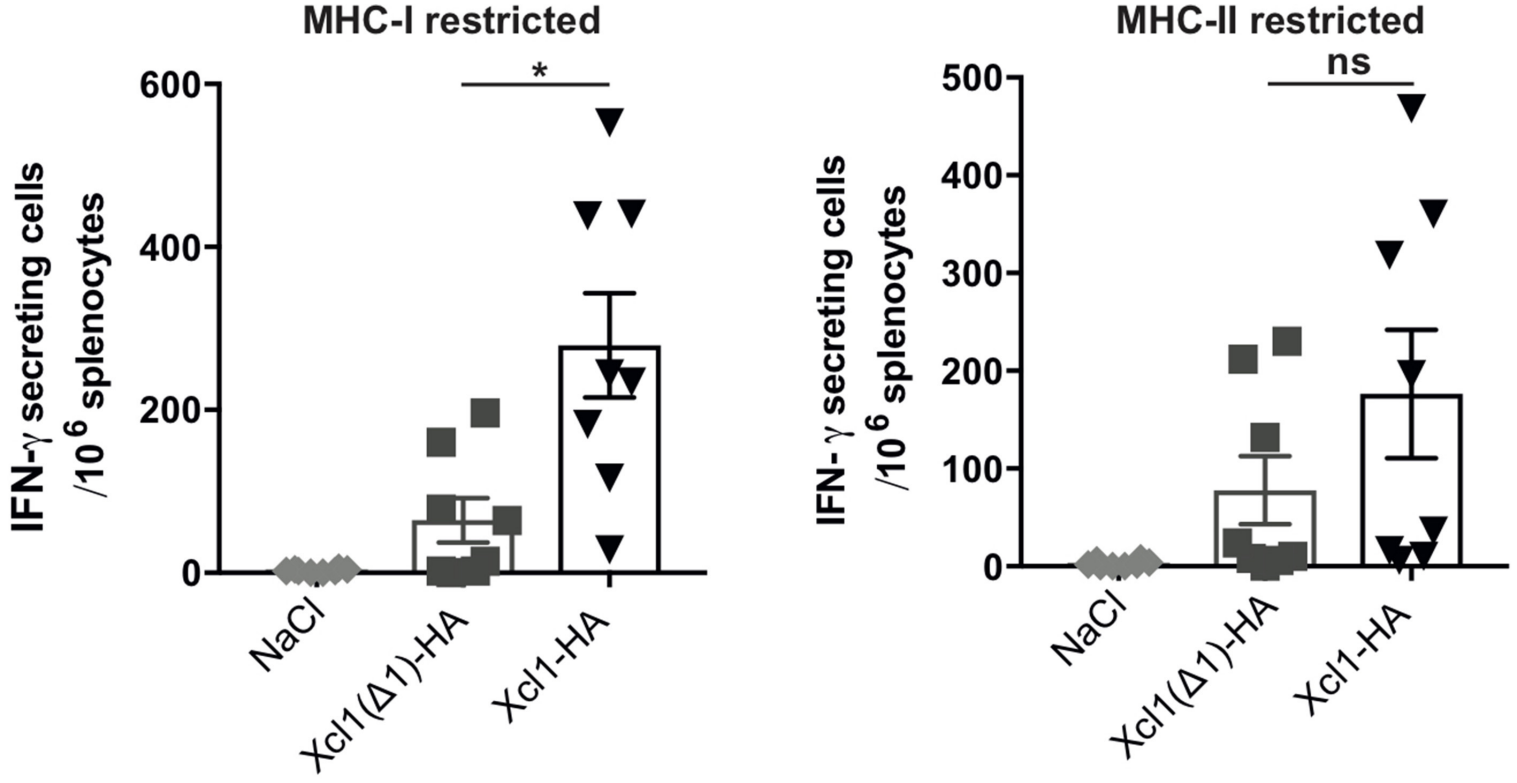
Xcl1(Δ1)-targeting gives lower CD8^+^ T cell responses. IFN-γ secretion by splenocytes in response to stimulation with the HA-derived peptides IYSTVASSL (MHC-I restricted) or HNTNGVTAACSHEG (MHCI-II restricted) 9 days after vaccination. Data shown are mean ± SEM. Data are pooled from two separate experiments, *n* = 8. Unpaired *t*-test (two-tailed) with welch correction, *p < 0.05.

Next, we investigated how lack of receptor activation and endocytosis influenced the T cell responses. BALB/c mice were sacrificed 9 days after DNA immunization with plasmids encoding Xcl1-HA or Xcl1(Δ1)-HA and splenocytes were analyzed for IFN-γ secretion by ELISPOT. Splenocytes were stimulated with the MHC-I restricted peptide IYSTVASSL or the MHC-II restricted peptide HNTNGVTAACSHEG to indicate CD8^+^ and CD4^+^ T cell responses, respectively. Vaccination with Xcl1(Δ1)-HA induced IFN-γ secreting T cells that responded to both peptides (Figure 4). However, in comparison to the WT Xcl1-HA immunized mice, the magnitude of the responses were lower, although the difference was only significant for splenocytes stimulated with the MCH-I peptide (Figure 4). These observations indicate that a lower degree of antigen internalization by cDC1s leads to reduced presentation on MHC-I and consequently a less potent induction of CD8^+^ T cells.

### Xcl1(Δ1) Targeting Increases the Frequency of HA Specific GC B Cells

**Figure 5:**
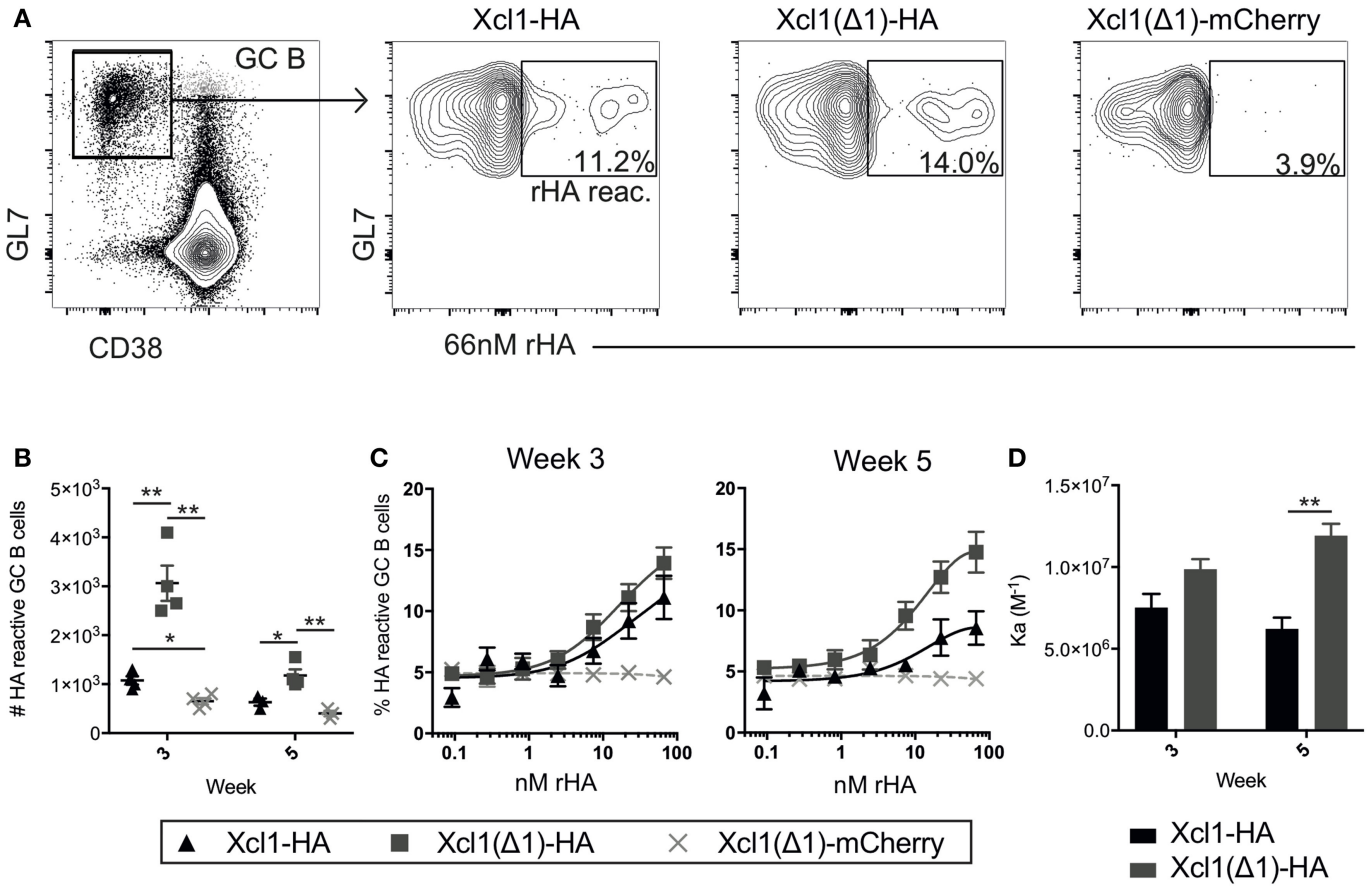
Xcl1(Δ1)-targeting results in improved GC B cell responses. **(A)** GC B cells were identified as CD3^−^B220^+^CD38^low^GL7^+^ and gated GC B cells were stained with a recombinant HA probe (66 nM). HA reactive GC B cells were detected as indicated in the representative data. **(B)** Absolute numbers of HA reactive GC B cells from iliac and inguinal lymph nodes were detected at weeks 3 and 5 after vaccination. **(C)** Titration curves of GC B cells with the HA probe indicate the avidity of the population as high affinity clones can bind antigen at lower concentrations. **(D)** Affinity constants were determined from the dilution curves in **(C)**. **(B–D)** *n* = 4. Data shown are mean ± SEM. **(B)** Unpaired *t*-test (two-tailed). **(D)** Extra sum of square *F*-test. *p < 0.05, **p < 0.01.

Increased antibody responses after targeting antigen to Clec9A have been associated with increased germinal center (GC) B cell responses (32). Indeed, a similar increase in GC B cells has been observed when targeting antigen to MHC-II (33) or CD11c (11). To investigate the GC response induced by Xcl1(Δ1)-HA, BALB/c mice were DNA immunized once and draining lymph nodes (LNs) (iliac and inguinal) were harvested after 3 or 5 weeks (34). Antigen reactive GC B cells were identified as CD3^−^B220^+^CD38^low^GL7^+^cells that bound a recombinant HA probe in flow cytometry (Figure 5A) (35). Negative control mice were vaccinated with Xcl1(Δ1)-mCherry, to induce a GC reaction against an irrelevant antigen (mCherry). Correlating with the antibody data above, intradermal DNA immunization with Xcl1(Δ1)-HA induced a significant increase in the number of HA specific GC B cells compared to WT Xcl1-HA 3 weeks after immunization (Figure 5B). The number of HA reactive GC B cells declined in LN 5 weeks after immunization, although the numbers were still significantly higher in Xcl1(Δ1)-HA immunized mice compared to Xcl1-HA (Figure 5B). To test if Xcl1(Δ1)-HA immunization also influenced the avidity of the antibody response, antigen reactive GC B cells were stained with titrated concentrations of the HA probe (35). There was no significant difference in HA specific avidity after 3 weeks, but Xcl1(Δ1)-HA immunized mice induced GC B cells with higher avidity at week 5 compared to Xcl1-HA (Figures 5C,D).

### Xcl1(Δ1)-HA Immunization Improves Protective Antibody Responses

**Figure 6:**
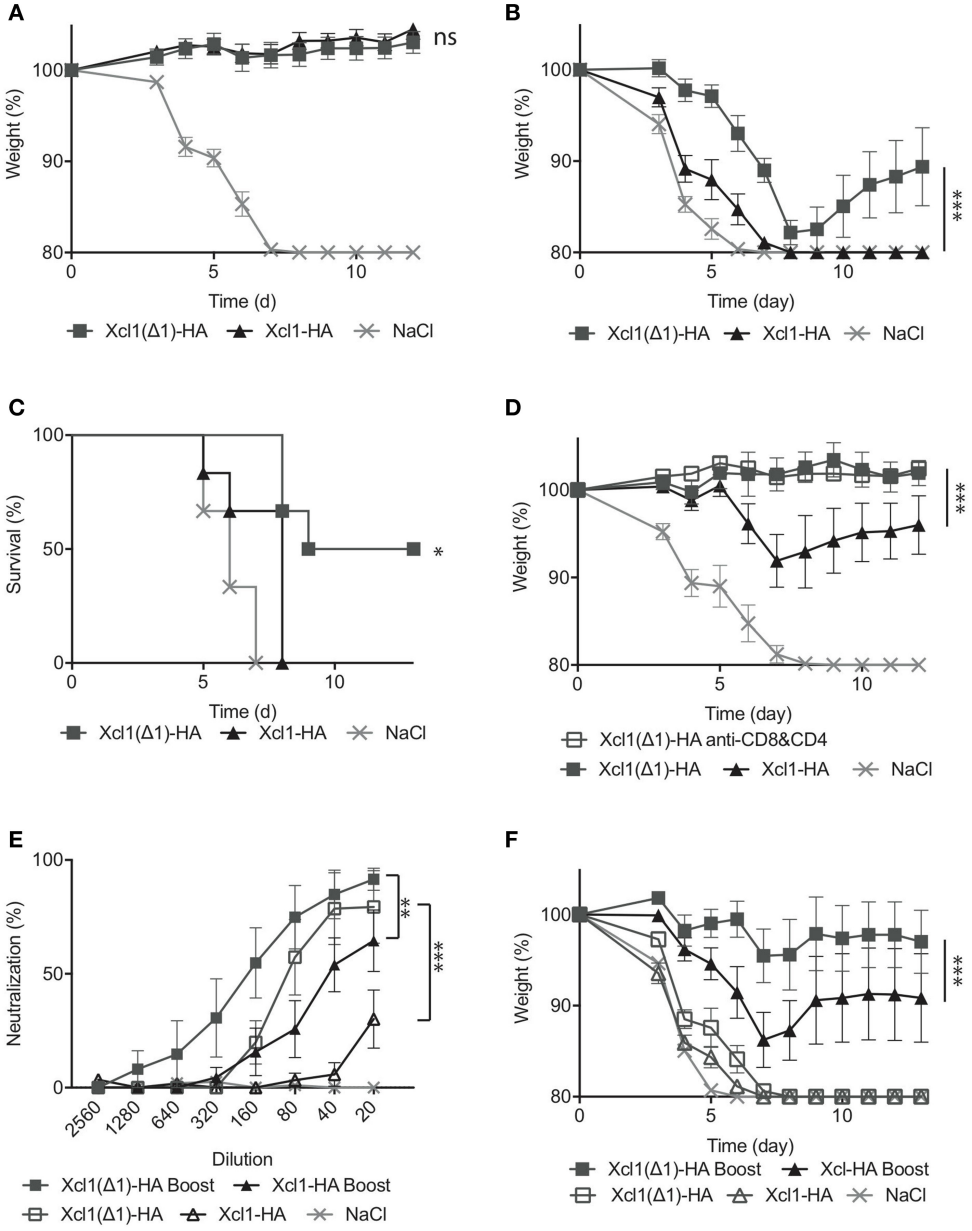
Xcl1(Δ1)-targeting results in protection from lethal PR8 challenge. **(A)** BALB/c mice were vaccinated as described in Figure 3, and challenged 6 weeks later with 5xLD50 of PR8 virus. Weight loss was monitored as a measure of disease progression. **(B)** Sera were harvested 6 weeks after vaccination, and transferred to naïve mice. The following day, the recipient mice were challenged with a 2,5xLD50 of PR8. **(C)** Survival plot of the mice presented in **(B)**. **(D)** 12 weeks after vaccination, mice received either anti-CD8 and anti-CD4 depleting mAbs, or isotype matched mAbs 3 days before, the day of, and 3 days after challenge with 5xLD50 of PR8. As in **(B)**, weight loss was monitored as a measure of disease progression. **(E)** Microneutralization was done with different dilutions of sera from vaccinated mice. **(F)** Serum transfer to naïve mice was done 6 weeks after the first immunization, 3 weeks after the second for boosted mice and mice were challenged with a 5XLD50 dose of PR8. **(A)** *n* = 12 for vaccine groups, *n* = 6 for NaCl group. **(B–F)** *n* = 6. **(A,B,D–F)** Data shown are mean ± SEM. Two-way ANOVA with Tukey's multiple comparisons test, **(C)** Mantel-Cox. *p < 0.05, **p < 0.01, ***p < 0.001.

Next, the protective efficacy of the Xcl1(Δ1)-HA vaccine was evaluated by immunizing BALB/c mice once and subsequently challenging them with 5xLD50 influenza A PR8 virus after 6 weeks. Both Xcl1(Δ1)-HA and Xcl1-HA induced full protection and minimal weight loss during the course of the infection (Figure 6A). We have previously observed that passive immunization with serum from Xcl1-HA immunized mice confers poor protection against viral challenge (9). In order to evaluate the protective abilities of the antibodies induced by Xcl1(Δ1)-HA, sera were harvested 6 weeks after immunization and transferred to naïve mice before challenge with a lethal dose of PR8. Xcl1(Δ1)-HA immunized mice demonstrated significantly reduced morbidity and increased survival compared to Xcl1-HA (Figures 6B,C). The WT Xcl1 group rapidly lost weight and all mice had to be euthanized by day 8. In fact, mice passively immunized with WT Xcl1 barely outperformed the negative control, demonstrating a significant increase in antibody contribution to protection for Xcl1(Δ1)-HA vaccinated mice (Figures 6B,C).

To evaluate a more long-term protection, we challenged mice 12 weeks after a single immunization and observed that while the protection afforded by WT Xcl1 targeting had waned by this time point, displaying weight loss after infection, Xcl1(Δ1) targeting still provided complete protection (Figure 6D). We also included a Xcl1(Δ1) group that received anti-CD4 and anti-CD8 antibodies resulting in depletion of T cells. The other vaccinated groups were injected with equal amounts of isotype matched antibodies, while depletion was confirmed by FACS analysis (Supplementary Figure 1D). Depletion of T cells had no effect on the protection provided by the Xcl1(Δ1) targeted vaccine, further attributing the observed protection to the antibody response (Figure 6D). To further investigate the difference in the quality of the antibody responses induced by the two vaccines, we performed a neutralization assay and observed that Xcl1(Δ1) targeting induced higher levels of neutralizing antibodies as compared to WT Xcl1 after a single immunization (Figure 6E). Next, to test if the protective antibody responses obtained after Xcl1(Δ1)-HA immunization could be boosted, we included mice that were immunized twice with 25 μg of WT Xcl1- or Xcl1(Δ1)-HA encoding plasmids 3 weeks apart. Boosting elevated the neutralizing ability of the antibody responses for both WT and Xcl1(Δ1), however Xcl1(Δ1) still gave higher levels of neutralizing antibodies as compared to WT Xcl1 after boost (Figure 6E).

Finally, sera from boosted mice were transferred to naïve mice, which were challenged with 5xLD50 PR8, a higher dose of virus than in the previous serum transfer experiment. At this dose, both un-boosted groups succumbed to the infection within 8 days. In contrast, both boosted groups performed significantly better. Again, the Xcl1(Δ1)-HA immunized group lost significantly less weight compared to the WT Xcl1-HA immunized group (Figure 6F), and 5/6 mice survived challenge compared to 3/6 for the WT, further confirming the beneficial effect of Xcl1(Δ1) targeting on antibody induction over WT Xcl1.

In summary, Xcl1(Δ1)-HA was superior to WT Xcl1-HA in inducing protective antibody responses in mice, an effect that was conserved after boosting.

## Discussion

Here, we identify a murine Xcl1 mutant (Xcl1(Δ1)) that maintains binding to the Xcr1 receptor, but does not induce activation and endocytosis. Immunization with an Xcl1(Δ1)-HA fusion vaccine enhanced antibody responses compared to a WT Xcl1-HA, as determined by higher IgG titers, increased numbers of GC B cells and improved protection in serum transfer experiments. In order to generate a murine Xcl1 mutant that bound, but did not activate the Xcr1 receptor, we evaluated a number of amino acid substitutions based on sequence comparison of human XCL1 and murine Xcl1. However, murine Xcl1 mutants containing alanine, or the human XCL1 aa, in position 8 and/or 9 all behaved as WT murine Xcl1 in terms of binding and endocytosis (Figures 1C,D). Consequently, aa positions 8 and 9 do not seem to play crucial roles in binding or activation in murine Xcl1. In contrast, removal of the valine in position 1 of the mature Xcl1 chemokine resulted in a mutant that retained binding but did not induce endocytosis of the Xcr1 receptor. The murine Xcl1(Δ1) mutant was based on a study by Tuinstra et al. where they observed that human XCL1(Δ1) failed to induce Ca^2+^-flux in XCR1^+^ cells (24). Our observations indicate that the murine Xcl1(Δ1) mutant behaves similarly. Considering that the valine in position 1 is largely conserved in Xcl1 in all mammals (36), it is likely that the Xcl1(Δ1) vaccination approach can be translated to a number of other species.

The experimental data presented here support our previous claim that targeting antigen to Xcr1^+^ cDC1s in absence of receptor mediated endocytosis can enhance antibody responses (9). While our previous study was performed using human XCL1 and XCL2 in a murine model, the results presented here are obtained using a murine chemokine as a targeting moiety. This suggests that our previous results are not due to the use of a foreign chemokine, which could contain helper epitopes that enhance immunogenicity. Indeed, since the Xcl1(Δ1) mutant only lacks one aa it is also unlikely that it would be immunogenic or break tolerance toward WT Xcl1. There are several conceivable scenarios in which an endocytosis deficient vaccine could result in improved antibody responses. As shown by Tam et al., optimally matching the antigen availability to the kinetics of the GC response is important for inducing good antibody responses (37). DNA vaccination would continually supply fresh antigen to the draining lymph nodes for a period of time, which is the case for the two vaccines in this study. However, the endocytosis deficient Xcl1(Δ1) would avoid intracellular degradation and remain intact, resulting in more antigen being available for B cells at any given time. Also, Xcl1(Δ1) targeting could result in antigen being present on the surface of cDC1s for a longer time, possibly resulting in activation of B cells entering lymph nodes through high endothelial venules (38). Available antigen on the surface of APCs may mimic immune complexes, known to increase BCR mediated activation of B cells (39), and it is conceivable that the endocytosis deficient vaccine results in greater synapse formation between the APC and the B cell which may result in improved responses (40). The above-mentioned scenarios all fit well with the observation that the endocytosis deficient vaccine gives lower CD8^+^ T cell responses. However, it is not yet clear to what extent the Xcl1(Δ1) remains on the surface of the cDC1 *in vivo*. It is possible that the weaker interaction between Xcr1 and Xcl1(Δ1) compared to the WT Xcl1, results in a higher off-rate of Xcl1(Δ1) resulting in higher antigen availability for the B cells. In any case, it is clear from our results that the nature of the interaction between vaccine proteins and receptors on DCs can affect the induced immune response, and that this interaction can be manipulated to induce the desired immune response against a DC targeted antigen. Importantly, the specific modification presented here, namely the removal of a single amino acid from the targeting chemokine should easily be translatable to larger animals, and has already been shown to result in loss of receptor activation for human XCL1 (24). The increased GC reaction, augmented antibody levels and protective abilities seen with the Xcl1(Δ1) fusion vaccine are of great interest for the development of many vaccines (41), especially where induction of high titers of neutralizing antibodies are of particular interest, such as influenza or malaria.

## Ethics Statement

All *in vivo* studies were pre-approved by the Norwegian Animal Research Authority, and performed in compliance with their guidelines.

## Author Contributions

AG, EF, TA, and VS-G generated reagents and performed experiments. AG, EF, and BB designed the experiments and conceptualized the study. AG, EF, and TA wrote the manuscript. All authors reviewed and approved the manuscript.

### Conflict of Interest Statement

The TTO office of Oslo University and Oslo University Hospital has filed several patents on Vaccibodies on which BB is an inventor. BB is head of the scientific panel of the Vaccibody Company and holds shares in the company. The remaining authors declare that the research was conducted in the absence of any commercial or financial relationships that could be construed as a potential conflict of interest.
