# Supplementary material for: Endocytosis Deficient Murine Xcl1-Fusion Vaccine Enhances Protective Antibody Responses in Mice
Source: Front Immunol. 2019 May 17;10:1086. doi: 10.3389/fimmu.2019.01086 (PMC6533920; doi:10.3389/fimmu.2019.01086)

Supplementary information

Endocytosis deficient murine Xcl1-fusion vaccine enhances protective antibody responses in mice.

Arnar Gudjonsson[1], Tor Kristian Andersen[1], Vibeke Sundvold-Gjerstad[3], Bjarne Bogen[1,2,*], and Even Fossum[1,*]

[1]K.G. Jebsen Centre for Influenza Vaccine Research, Institute of Immunology, University of Oslo and Oslo University Hospital, 0450 Oslo, Norway.

[2]Centre for Immune Regulation, Institute of Immunology, University of Oslo and Oslo University Hospital, 0027 Oslo, Norway.

[3]Department of Molecular Medicine, Institute of Basic Medical Sciences, University of Oslo, 0317 Oslo, Norway.

[*] Correspondence and requests for materials should be sent to:

Even Fossum or Bjarne Bogen

Institute of Immunology

Oslo University Hospital

Songsvannveien 20

0027 Oslo,

Norway

Phone: +47 23 07 30 45

Fax: +47 23 07 35 10

Even.fossum@rr-research.no

Bjarne.Bogen@medisin.uio.no

SUPPLEMENTARY FIGURE.

(**A**) Schematic representation of the vaccibody molecule containing a targeting unit (Xcl1 or Xcl1(Δ1)), a dimerzation domain and antigenic unit (hemagglutnin or mCherry). (**B**) BM-derived Flt3L-induced DCs were defined as CD45R/B220^{-}CD11c^{+} and further divided into CD24^{+} (cDC1) or CD11b^{+} (cDC2). (**C**) Splenic DCs were defined as CD3e^{-}CD19^{-}CD49b^{-}Ly6G^{-}MHC-II^{+}CD11C^{+} and further split into CD24^{+} (cDC1) or CD11b^{+} (cDC2). (**D**) Spleens were harvested from mice three days after i.p. injection with 200 μg of depleting or isotype matched antibodies. T cells were defined as CD45R/B220^{-}CD3^{+}.

Supplementary Figure

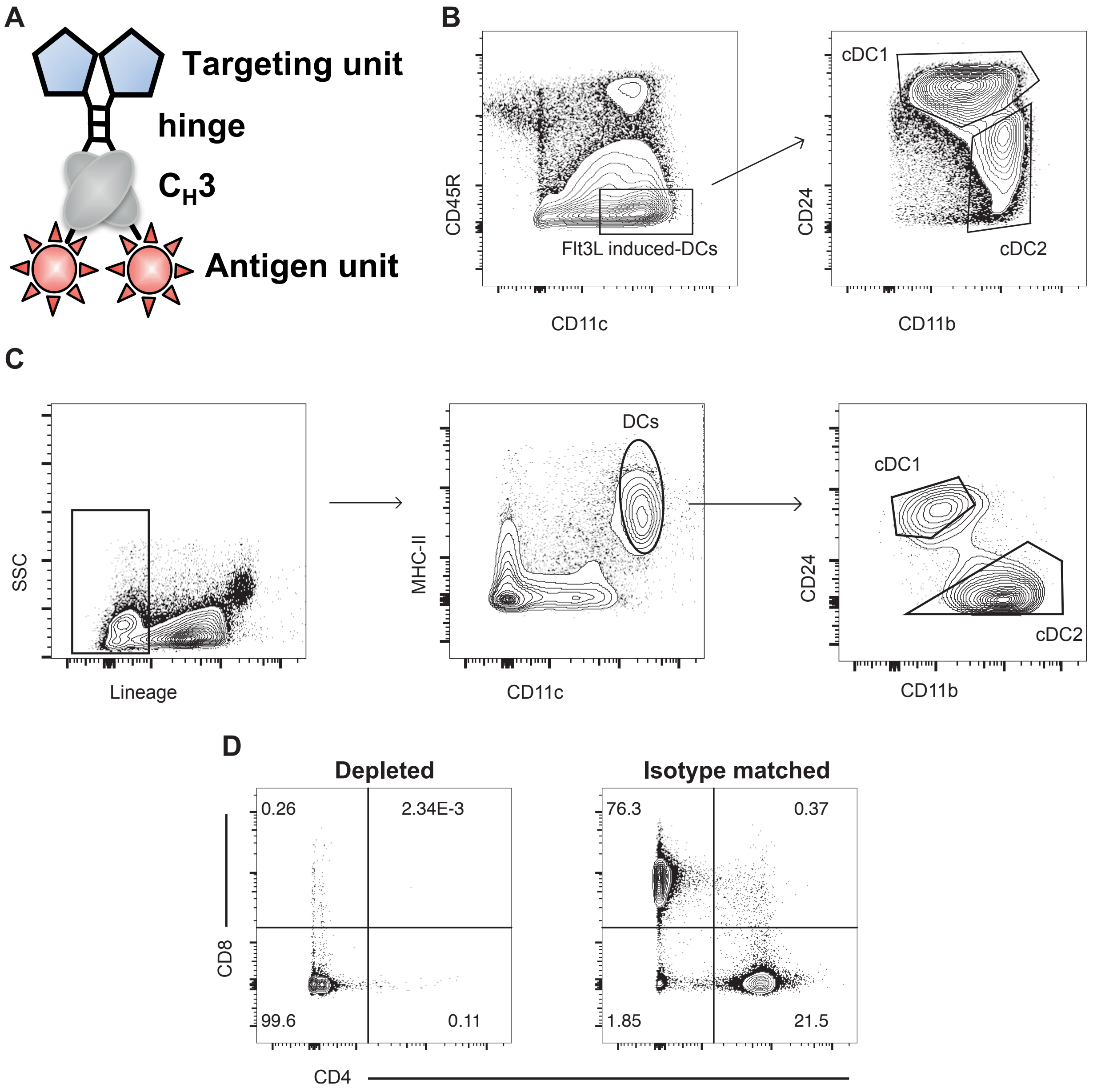

Supplement: Supplementary file 1 [file Data_Sheet_1.pdf]
